# Torts of Hospitals

**Published:** 1909-05-08

**Authors:** 


### Medico-Legal Points.

### TORTS OF HOSPITALS.

The case of *Hillyer* v. *The Governors of St. Bartholomew's Hospital*, which was tried recently before Mr. Justice Grantham and a special jury, raised a question of considerable importance to the governors of hospitals and other charitable institutions. The plaintiff, a member of the medical profession, sued the Governors for damages for personal injuries suffered by him whilst undergoing an examination under anæsthetics. It will be remembered that the plaintiff, who had entered the hospital for gratuitous treatment and examination, said that by reason of the negligence of the defendants or their servants his left arm came into contact with the hot-water tins used for heating the operating-table, and that as a consequence he was paralysed in that arm, while the right arm was also injured. Defendants denied negligence on their part, and asserted, moreover, that the relation of master and servant did not exist between them and those engaged in the examination, and that they were not, therefore, responsible in law. After hearing most of the evidence on behalf of the plaintiff, Mr. Justice Grantham said that, on the pleadings, he considered it would be impossible for the plaintiff to get a verdict. The plaintiff's case had been heard, and he had produced no evidence to lead his lordship to change his original opinion that the plaintiff could not succeed. The causes of the plaintiff's injuries remained as obscure as they were before his evidence was given, and he had not even proved a case of negligence. In his view, the governors would not be liable for the negligence of the staff if proved. There would be judgment for the defendants, with costs.

The cases in which actions have been brought against hospitable and other institutions for the alleged negligence of their medical officers have not been numerous, and apparently in no cases have they been successful. In the case of *Evans* v. *Mayor of Liverpool* (1906, 1 K. B. 160) the defendants, a local authority, acting under the provisions of the Public Health Act, 1875, provided for the use of the inhabitants of their district a hospital for the reception of persons suffering from infectious diseases. A visiting physician, who was a competent medical practitioner, was appointed to the hospital, and acted under the general directions of the hospital committee of the defendants, the rules providing (*inter alia*) that he should be responsible for "the treatment of the patients from the beginning to the end of their stay, and also for their freedom from infection when discharged." A son of the plaintiff was treated in the hospital while suffering from a mild attack of scarlet fever, and was ultimately discharged by the visiting physician while still in an infectious condition, and under circumstances which a jury found to amount to a want of reasonable skill and care in and about the discharge; for after the boy had returned home he communicated the disease to three other children of the plaintiff. The plaintiff then sued the defendants to recover the expense to which he had been put in regard to the illness of his other children owing to the premature discharge of his son from the hospital by the visiting physician. It was held by Mr. Justice Walton that the plaintiff was not entitled to recover, for the legal obligation of the defendants extended only to the provision of reasonably skilled and competent medical attendance for the patients, which they had discharged, and that there was no absolute undertaking or obligation on their part that no patient should be discharged by the visiting physician while still in a condition which might cause infection.

In *Hall* v. *Lees* (1904, 2 K. B. 602) the defendants were an association whose object was to provide for the supply of duly qualified nurses to attend on the sick in a certain neighbourhood. The association for that purpose appointed and paid salaries to nurses, for whose services they made charges to persons on whose application the nurses were supplied. The association printed rules and regulations with regard to the duties of their nurses and other matters with a view as well to the protection of the nurses as to ensuring their efficiency while engaged in nursing. These regulations provided for the exercise of certain supervision over the nurses by a superintendent appointed by the association; but, with regard to the work of a nurse while engaged in nursing a patient, it was provided (*inter alia*) that, while so engaged, she should not absent herself from duty without the permission of the patient's friends, and that she should implicitly follow the instructions of the patient's medical man. A form, which was sent out by the association upon supplying nurses, indicated to the person applying for the nurse that, while engaged in nursing the patient, the nurse was to be regarded as employed by that person. Two nurses were supplied by the association for the purpose of nursing the female plaintiff through an operation which was about to be performed upon her. An injury was occasioned through the negligence of the nurses or one of them. It was held by the Court of Appeal that, upon the true construction of the documents in relation to the supply of nurses, the contract of the association was not to nurse the female plaintiff through the agency of the nurses as their servants, but merely to procure for her duly qualified nurses, and that the nurses were not, in nursing the female plaintiff, acting as the servants of the association, and therefore the defendants were not liable in respect of negligence of the nurses supplied by them.

The liability of the board of governors or committee of a hospital or dispensary to any patient treated there for injuries arising from the negligence of the surgeon or medical practitioner whom they have appointed as their medical officer has been exhaustively discussed in America.

In the case of *McDonald* v. *Massachusetts General Hospital* (120 Mass. 432) the plaintiff, who had sustained a fracture of the thigh-bone by falling from a building, was brought to the defendant hospital, where he received gratuitously the surgical and medical care, attendance, and nursing which the hospital afforded its patients, occupying a free bed and having all the expenses attending his injuries borne as a charity by the defendant. The bone was set, in the first instance, by the house pupil, to which the plaintiff objected at the time, as he preferred the resident physician. Both the house pupil and a visiting surgeon afterwards attended the case. The plaintiff claimed that the bone was not properly set, and laid damages therefor. It was in evidence that the house pupil had been well recommended, and that the visiting surgeon was one of several who were selected by the trustees as visiting surgeons (being men of skill and training), and who gave their services without pay. The judge in the Superior Court ruled that "even if the plaintiff should prove that the fractured bone was not properly set, in consequence of the incompetency of the house pupil, or his negligence, or that of the attending physician, the plaintiff was not entitled to recover." The jury found for the defendant, but the Supreme Court of the Commonwealth ordered judgment on the verdict. This case was decided, partially at any rate, on the authority of *Holliday* v. *St. Leonard's, Shoreditch* (11 C. B. (N.S.) 192), which, after the remarks of Mr. Justice Blackburn in *Foreman* v. *Mayor of Canterbury* (L. R. 6 Q. B. 218), must be considered as overruled.

In the subsequent case of *Glavin* v. *Rhode Island Hospital* (34 Am. R. 675) the same point again came up for decision. In that case it appeared that a circular-saw had left but two fingers upon the plaintiff's right hand, and that he applied to the defendant hospital for care and attendance, for which he was to pay a reasonable compensation. He was received by the superintendent, who placed him in charge of a hospital *interne*, who treated the injuries so unskilfully that what the saw failed to do the knife was successful in accomplishing, and the whole arm was amputated. It was shown that no one of the regular, experienced surgeons of the hospital came to see the plaintiff for seventeen hours after he was received in the hospital. The defendant introduced evidence showing that the hospital was administered as a charity, its income being derived from endowments and voluntary contributions; that the physicians attendant on the hospital gave their services without compensation, and the *internes* likewise, save that the *internes* had their board and lodging at the hospital. The charge made to the plaintiff (*i.e.* the "reasonable compensation" above referred to) was designed only to cover board, washing, warmth, and the services of nurses and ward tenders. At the trial a verdict was directed for the defendant. But on the hearing of the plaintiff's petition for a new trial, the full Bench granted the same.

In *Higgins* v. *McCabe* (126 Mass. 13) the defendant was employed as a midwife at the confinement of the plaintiff's mother. While so employed she undertook to treat the plaintiff, at whose birth she had assisted, intimating that she had cured others similarly affected (*i.e.* with inflammation of the eyes). Her kindly proffered services resulted in the total blindness of the plaintiff. The form in which the case went to the full Bench embraced the question of the defendant's liability on account of such services, which were rendered gratuitously and resulted disastrously. It will be noticed that this woman did no more than a stranger might have done. She was hired to assist the mother; she was not employed to tend the diseases of the child. She offered to do merely a friendly act, and she was not an oculist. And so the Court well ruled that, in the absence of any evidence as to whether skill as an oculist was embraced in a midwife's duties, the action could not be maintained.

A physician must apply the skill and learning which belongs to his profession, but a person who, without special qualifications, volunteers to assist the sick, can at most be only required to exercise the skill and diligence usually bestowed by persons of like qualifications under like circumstances. To have held otherwise would have been to charge responsibility in damages upon all who make mistakes in the performance of kindly offices for the sick. But a physician certainly should apply the usual care demanded of him, considering the present conditions of the profession, and the skill employed in cases like the one in which he is called to act. Now while, as a general rule of law, it may be said that a gratuitous agent is liable for gross negligence only, yet the Courts are fast dismissing the consideration of the various degrees of care required by parties, and confine themselves to the one consideration—whether due care was used under all the circumstances in the case. Certainly it would be monstrous for a physician to plead his fee against his own negligence.

In *Glavin* v. *Rhode Island Hospital* the Chief Justice said: "It is quite conceivable that a corporation might not agree to do more than furnish hospital accommodations, leaving the patient to find his own physician or surgeon. In such a case the corporation would plainly not be liable for the torts of the physicians or surgeons, for in such a case they would not be its servants, and it would not have assumed any responsibility in their selection. But that is not this case. Here the physicians or surgeons are selected by the corporation or the trustees. But does it follow from this that they are the servants of the corporation? We think not. If A out of charity employs a physician to attend B, his sick neighbour, the physician does not become A's servant; and A, if he has been duly careful in selecting him, will not be answerable to B for his malpractice. The reason is that A does not undertake to treat B through the agency of the physician, but only to procure for B the services of the physician. The relation of master and servant is not established between A and the physician, and so there is no such relation between the corporation and the physicians and surgeons who give their services at the hospital. It is true the corporation has power to dismiss them, but it has this power not because they are its servants, but because of its control of the hospital where their services are rendered. They would not recognise the right of the corporation, while retaining them, to direct them in their treatment of patients. But though the relation of master and servant cannot be said to exist between the hospital and the physicians and surgeons attending on it, the hospital does nevertheless assume a responsibility in that it uses its own judgment, or that of its trustees, in selecting them, and impliedly therefore undertakes to exercise reasonable care to get such as are skilful and trustworthy in their profession. A patient has a right to rely on the exercise of such care, and consequently if, through the neglect of the hospital to exercise it, he receives an injury, he is entitled to look to the hospital for indemnity unless the hospital enjoys some extraordinary exemption from liability."

In New Zealand the point has been decided by the Court of Appeal as in *Glavin's Case*, and on the authority of the reasoning thereon. In *The District of Auckland Hospital and Charitable Aid Board* v. *Lovett* (10 N. Z. L. R. 597) the appellant board was a board incorporated by the Hospitals and Charitable Institutions Act, 1885, and having under that Act the superintendence and control of the A Hospital. C, the medical superintendent of the hospital, was appointed by the board at a salary, and was by the terms of his contract with the board to have complete control over all departments of the hospital, and the treatment of the patients therein, and was to be responsible to the board alone. The respondent, who was treated surgically by C in the hospital, afterwards brought an action against the board for injury alleged to have been caused by negligence and improper treatment by C. It was admitted that the board had exercised due care in appointing C, that he was duly qualified, that there was nothing unsatisfactory in his previous career, and that nothing had occurred in the hospital to give the board reason to doubt his competence. The treatment of the respondent was purely gratuitous and eleemosynary, a small charge for board only being made. It was held that the liability of his board was the same as that of a private person doing the same things would have been, there being nothing in the Hospitals and Charitable Institutions Act, 1885, to suggest that it should be less; but that there was no liability under the circumstances, the relationship of master and servant not existing between the board and C, the medical superintendent.

